# Erectile function after permanent 125I prostate brachytherapy for localized prostate cancer

**DOI:** 10.1186/2051-4190-23-2

**Published:** 2013-08-29

**Authors:** Patrice Njomnang Soh, Boris Delaunay, Matthieu Thoulouzan, Frederic Jonca, Jean Marc Bachaud, Martine Delannes, Michel Soulie, Eric Huyghe

**Affiliations:** Department of Andrology, University Hospital Paule de Viguier, Paul Sabatier University, EA 3694 Toulouse, France; Department of Urology, University Hospital of rangueil, Toulouse, France; Clinique Ambroise Paré, Toulouse, France; Institut Claudius Régaud, Toulouse, France; Département d’Urologie CHU Rangueil, 1 av. Jean Poulhès, TSA 50032, 31059 Toulouse Cedex 9, France

**Keywords:** Erection, Brachytherapy, Sexual function, Questionnaire, Carcinoma of the prostate, Érection, Curiethérapie, Function sexuelle, Questionnaire, Cancer de la prostate

## Abstract

**Background and purpose:**

To analyze erectile function in men treated by prostate brachytherapy (PB) for localized prostate cancer.

**Material and methods:**

Of a series of 270 sexually active men treated by PB, 241 (89%), mean age 65 yr (range, 43–80 yr), participated in a study on erectile function that was evaluated using the International Index of Erectile Function 5-item (IIEF-5) questionnaire before implantation and by postal survey after a mean follow-up of 36 months (range, 6–70 months).

**Results:**

After PB, 27 patients (11%) had no erectile dysfunction (ED), 36 (15%) had mild ED, 58 (24%) had mild to moderate ED, 24 (10%) had moderate ED, 53 (22%) had severe ED and 43 (18%) were not sexually active. In patients with a preimplant IIEF score >12 (cut-off for intercourse with penetration), 73% had a deterioration of erectile function by at least one class after PB. Risk factors for ED after PB were age, preimplant IIEF score and prostate volume. Median time to ED onset was 16 months and was shorter with androgen deprivation (p = 0.007), diabetes (p = 0.03) and age over 55 (p = 0.01).

**Conclusions:**

Following PB, the majority of patients progressively develop or major ED after a free interval that may last several months.

**Support:**

Ligue Nationale contre le Cancer, France

## Résumé

**Objet:**

Etude de la fonction érectile chez les hommes traités par curiethérapie pour un cancer localisé de la prostate.

**Matériels et Méthodes:**

A partir de 270 hommes sexuellement actifs, traités par curiethérapie, 241 (89%), moyenne d’âge 65 ans (entre, 43–80 ans), acceptaient de participer à l’étude de la fonction érectile après curiethérapie. Cette étude menée par le questionnaire validé IIEF 5 (International Index of Erectile Function 5-item), évaluait la fonction érectile avant curiethérapie, et en moyenne 36 mois (entre 6-70mois) après la curiethérapie de prostate. L’enquête était faite par envoi postal.

**Résultats:**

Après la curiethérapie, 27 patients (11%) n’avaient pas de dysfonction érectile, 36 (15%) avaient une dysfonction très modérée, 58 (24%) entre très modérée et modérée, 24 (10%) modérée, 53 (22%) avaient une dysfonction érectile sévère et 43 (18%) n’étaient plus sexuellement actifs. Parmi les patients ayant un score IIEF avant curiethérapie >12 (score moyen permettant une pénétration pendant l’acte sexuelle), 73% avait une détérioration de leur fonction érectile d’au moins une classe IIEF. Les facteurs de risque de la dysfonction érectile après curiethérapie étaient: l’âge, le score IIEF avant curiethérapie et le volume de la prostate.

La période moyenne pour déclencher une dysfonction érectile après curiethérapie était de 16 mois. Cette période se réduisait lorsque les patients étaient sous hormonothérapie (p = 0.007), avaient du diabète (p = 0.03) et étaient âgés de plus de 55 ans (p = 0.01).

**Conclusions:**

Après curiethérapie, la majorité des patients développaient progressivement une dysfonction érectile plusieurs mois après la curiethérapie.

## Introduction

In Europe as well as in North America, carcinoma of the prostate (CaP) is the most frequent cancer in men before lung cancer [1, 2]. Moreover, its incidence has been increasing during recent decades and continues to rise. Thanks to efforts toward early diagnosis, CaP is mostly diagnosed at an early stage. Until now, comparison of different treatments according to long-term oncological results has been controversial [3], putting functional aspects at the forefront in decision counseling. Erectile dysfunction (ED) is a major preoccupation for CaP patients, especially younger ones. In a series of men treated for localized CaP 12 to 24 months previously, Bokhour et al. found that ED affected not only the quality of sexual intimacy, but also everyday interaction with women, sexual imaging and fantasy life, and manliness perception [4]. A meta-analysis of 54 articles concluded that the predicted probability of maintaining erectile function was 0.76 after prostate brachytherapy (PB), 0.60 after PB plus external beam radiotherapy, 0.55 after external beam radiotherapy, 0.34 after nerve-sparing radical prostatectomy and 0.25 after standard radical prostatectomy [5]. However none of the articles analyzed were randomized controlled trials, some of the studies did not reflect current standards of treatment and none of the PB studies had a follow-up longer than 2 years. The latter point is crucial in view of the possibility of delayed deterioration of erectile function following radiation treatments [6, 7]. However, so far, observations of changes over time of the prevalence of ED after PB remain controversial. Stock et al. found that the proportion of potent men decreased over time following PB (79% at 3 years, 59% at 6 years) [8]. Valicenti et al. observed that after PB, one patient on two had ED, but that dysfunction resolved within one year after treatment [9]. Finney et al. found no significant correlation between the proportion of men with ED and time since implantation, with a median follow-up of 2.5 years [10].

The recommendations of the American Brachytherapy Society illustrate this uncertainty, concluding that both severity and duration of morbidities after treatment may vary from one patient to another [11].

This pleads for a better knowledge of ED after PB, in order to:

1. provide patients with fuller information about their long-term risk of ED,
2. develop more cost-effective interventions
3. help in the development of new protocols of clinical research on ED in this population.

The primary goal of the present study was to analyze the occurrence of ED after PB and its associated risk factors in a series of sexually active men treated by permanent 125I brachytherapy.

## Patients and methods

### Patients

During the study period (2000–2007), the Institut Claudius Régaud cancer center, Toulouse was the unique center in the Midi-Pyrénées region (South West France) to perform prostate brachytherapy. Therefore, all the patients’ candidate for this modality of treatment were referred by urologists to this center.

Inclusion criteria were CaP stage T1c/T2N0M0, Gleason score ≤ 7 (3 + 4), PSA < 10 ng/ml, prostate volume < 50 cc, no history of trans-urethral resection of the prostate, and index of prostatic symptom score (IPSS) < 8.

Between 2000 and 2006, 316 consecutive patients were implanted with radioactive iodine 125I seeds using a real-time ultrasound-based planning technique (real-time computer-assisted dosimetry with dynamic seed localization performed in the operating room). The prescribed brachytherapy dose was 160.5 Gy. According to the post treatment dosimetry, the mean dose to 90% of the outlined prostate volume (D90) was 160.5 Gy and the mean percentage of prostate volume receiving at least 100% of the prescribed dose (V100) was 88%. Sixty-three (26%) patients received neoadjuvant androgen deprivation therapy for a mean of 4 months (range 2 to 24 months). No patient received supplemental external beam radiation therapy or surgery potentially damaging to erectile function. A questionnaire on sexual activity (including the IIEF-5 questionnaire) was systematically completed by the patients before the beginning of treatment (during the preimplant consult).

### Survey

We performed our survey of sexual dysfunction in November 2006. All patients who were sexually active before PB were contacted by mail irrespectively of whether or not they had a committed partner. An introductory letter was mailed to the patients that included the questionnaire, the name of the research contact person and phone numbers for further information. Evaluations were completed by mail and a reminder letter was sent if questionnaires were not returned within four weeks. Anyway, each patient completed only one post-treatment questionnaire.

Of the 316 patients, 28 men who were not sexually active before PB, eight who needed a second implantation and 10 who had died (of causes unrelated to cancer) were excluded. After treatment, the 270 eligible patients were asked to participate in the survey of sexual dysfunction and were considered as agreeing to participate if they completed the questionnaire. Only 29 refused to take part (participation rate: 89%). Finally, we obtained information about sexual function before and after PB in 241 patients. We checked that there were no significant differences in demographic, medical and PB parameters between responders and non-responders (Table 1). Tables 2 and 3 summarize the characteristics of the population who participated in the study (n = 241) and their treatment parameters. The mean interval between the end of treatment and post-treatment survey was 36 months (range 6 to 70 months).

**Table 1:** **Comparison responders - non responders**

	***Responders***	***Non responders***	
Variables	(n = 269)	(n = 29)	P-value
*Age (year)*	66,4 ± 7,2	65,6 ± 7,9	0,67
*Pre-treatment index of prostatic symptom score (IPSS)*	4,1 ± 2,8	4,2 ± 2,8	0,75
*Pre-treatment PSA (ng/ml)*	6,7 ± 2,3	7,1 ± 2,4	0,32
*Pre-treatment prostate volume (cm3)*	34,2 ± 9,7	33,7 ± 9,6	0,89
*Gleason score (% points)*		0,52	
*< VII*	82	79	
*VII*	18	21	
*Clinical tumour stage (% points)*		0,43	
*T1C*	60	72	
*T2*	40	28	
*Neoadjuvant hormonal therapy (% points)*	27,5	38	0,28
*Follow-up (year)*	3,0 ± 1,5	2,6 ± 1,3	0,24
*D90 (minimal dose received by 90% of the prostate gland)*	159,8 ± 21,8	151,7 ± 21,7	0,08
U30* (minimal dose received by 30% of urethra)	215,5 ± 44,0	198,5 ± 40,0	0,06
V100* (percentage of the prostate volume receiving 100% of the prescribed dose)	87,7 ± 8,0	85,5 ± 7,6	0,09
V150* (percentage of the prostate volume receiving 150% of the prescribed dose.)	47,5 ± 14,6	43,0 ± 12,0	0,14
RV160* (surface of rectum receiving 160 Gy)	0,58 ± 0,76	0,42 ± 0,55	0,42
*Number of implanted seeds*	74,2 ± 11,7	72,1 ± 12,6	0,41
*Number of implanted needles*	21,2 ± 2,7	21,3 ± 3,0	0,87

* measurements 30 days after implantation.

**Table 2:** **Characteristics of the population (n = 241)**

		Mean ± SD (Min-max)
*Age (years)*		66 ± 7 (43 – 80)
*Pre-treatment PSA (ng/mL)*		6.6 ± 2.2 (1.2 - 12.6)
*Pre-treatment index of prostatic symptom score (IPSS)*		4.0 ± 2.8 (0 – 15)
*Pre-treatment prostate volume (cm* ^*3*^ *)*		36.3 ± 9.7 (14 – 67)
		N (%)
*Gleason score*	None	10 (4.2)
	< 6	43 (17.8)
	6	146 (60.6)
	7	42 (17.4)
*Stage*	T1C	147 (61)
	T2A	89 (37)
	T2B	5 (2)
*Diabetes*		19 (8)
*Vascular disorders*		46 (19)
*Obesity (Body mass Index > 2.8)*		22 (9)
*Contraindication to surgery*		25 (10)
Neoadjuvant hormonal therapy*		63 (26)

* LHRH agonists in 10 patients (15.9%), non-steroidal antiandrogens in 6 (9.5%), cyproterone acetate in 25 (39.7%), and complete androgen blockade in 22 (34.9%).

**Table 3:** **Prostate brachytherapy parameters (n = 241)**

***Number of implanted seeds***	
Mean ± SD	74.3 ± 11.6
Min-max	47-111
*Number of implanted needles*	
Mean ± SD	21.4 ± 2.7
Min-max	12-31
D90* (minimal dose received by 90% of the prostate gland)	
Mean ± SD	160.5 ± 22.2
Min-max	97.5-222
U30* (minimal dose received by 30% of urethra)	
Mean ± SD	215 ± 44.7
Min-max	14.1-395.8
RV160* (surface of rectum receiving 160 Gy)	
Mean ± SD	0.56 ± 0.76
Min-max	0-5
V100* (percentage of the prostate volume receiving 100% of the prescribed dose)	
Mean ± SD	88 ± 7.9
Min-max	61.8-99.5
V150* (percentage of the prostate volume receiving 150% of the prescribed dose)	
Mean ± SD	47.5 ± 14.5
Min-max	16.1-84.7

*: measurements 30 days after implantation.

### Measures

#### *Demographic and medical measures*

Sociodemographic data, disease and treatment characteristics were recorded prospectively.

#### *Measurement of erectile function*

The questionnaire on sexual function covered various aspects of sexual activity. Here we present only the results relating to erectile function. The questionnaire was similar to the pre-treatment inventory of sexual function in order to be able to compare changes over time.

In order not to bias the results due to the use of medications (notably PDE5 inhibitors), it was stressed in the introduction letter and in the questionnaire that the patients had to describe the quality of erection without any medication, and a special question focused on treatment for sexual dysfunction. All patients who declared using a medication (n = 36) were called by phone by a research assistant to verify their answer.

According to the recommendations of the American Brachytherapy Society [11], erectile function was assessed using the IIEF-5 questionnaire (French version) [12]. To analyze the severity of ED, we used the accepted classes: a score of 5 to 7 defined severe ED, 8 to 11 moderate ED, 12 to 16 mild to moderate ED, 17 to 21 mild ED, and a score above 22 no ED [12, 13].

### Statistical methods

Statistical analysis was done using STATA SE 8.2^®^ (Stata Corporation College Station, TX) and R version 2.3.1 software and a confidence interval of 95%. Descriptive statistics were performed for all studied variables with a normality test for all quantitative variables. Inter-group comparisons were performed using the chi-2 (or Fisher exact) test for qualitative variables and the Mann–Whitney test for quantitative variables. IIEF scores before and after PB were compared using the Wilcoxon test. As there were a large number of variables, we first performed factorial analysis to obtain a graphic representation to reveal potential interactions between variables. Associations between IIEF score and variables were sought with the Mann–Whitney test and Spearman’s correlation test. ED risk factors were studied by logistic regression analysis with adjustment for age, neoadjuvant androgen deprivation therapy and PSA level. Significant variables in univariate analysis (threshold at 10%) were analyzed by multivariate analysis

The log-rank test was used for inter-group comparisons. Cox regression was applied to determine the links between variables and occurrence over time of ED (hazard ratio), when the proportional hazards hypothesis was verified.

### Ethical approval

In accordance with French regulations about clinical research, it was not required to have agreement from an ethics committee for such an observational study of a validated treatment.

## Results

### Erectile function status

After implantation, patients had a significantly lower mean IIEF score than before implantation (13.2 +/- 7.5 vs. 19.1 +/- 5.5; p < 0.0001).

Before PB, 108 patients (45%) had no erectile dysfunction (ED), 65 (27%) had mild ED, 43 (18%) had mild to moderate ED, 17 (7%) had moderate ED, and 7 (3%) had severe ED. After PB, 27 patients (11%) had no ED, 36 (15%) had mild ED, 58 (24%) had mild to moderate ED, 24 (10%) had moderate ED, 53 (22%) had severe ED and 43 (18%) were not sexually active.

Of the 216 patients who had a score ≥ 12 before PB (cut-off for intercourse with penetration), 32 (15%) ceased to be sexually active after treatment. Of the 184 patients who continued to be sexually active, 46 (25%) remained in the same IIEF score class, 56 (30%) downgraded by one class, 78 (42%) by two or more classes, and 4 (2%) upgraded by one class.

Patients with an IIEF score <12 before PB had a significantly higher risk of becoming sexually inactive after PB (p < 0.001). In the severe ED group before PB, 50% became sexually inactive after PB.

### Risk factors for erectile dysfunction

#### *Factorial analysis*

Factorial analysis showed a strong association between the IIEF score after PB and age at implantation. An IIEF score < 7 after PB appeared to be linked to the presence of comorbid conditions (diabetes, obesity, history of cardiovascular disorders, contraindications to surgery) and/or neoadjuvant androgen deprivation therapy.

#### *Correlations*

The IIEF score after PB was correlated with age at implantation (r = 0.18, p = 0.013) and with the IIEF score before PB (r = 0.47, p < 0.001). On the other hand, it did not appear to be correlated with PB parameters (D90, V100, V150, RV160, U30), nor with the length of time between PB and evaluation. An affirmative response to the question « At the time you found that you had prostate cancer, was maintaining sexual activity of concern to you? (yes/no) » was associated with a higher IIEF score before and after PB (p = 0.0001, p = 0.0182).

#### *Logistic regression*

The results of logistic regression were adjusted for age, neoadjuvant androgen deprivation therapy and PSA level. Univariate analysis was performed for the following variables: age, IIEF score and IIEF classes before PB, existence of morning erection, concern about sexuality, neoadjuvant hormonal therapy, pre-treatment PSA level, IPSS score and prostate volume, history of diabetes, vascular disorders and obesity, contraindication to surgery, brachytherapy parameters D90, V100, V150, U30, RV160, number of implanted seeds and number of implanted needles. Table 4 summarizes significant risk factors for erectile dysfunction in univariate analysis. These were taken into consideration for multivariate analysis (Table 5). The variables significantly associated with the risk of ED after PB were age (OR = 1.06, 95% CI = 1.02–1.12), IIEF score before implantation (OR = 0.89, 95% CI = 0.84–0.95) and prostate volume (OR = 0.96, 95% CI = 0.93–0.99). It was noteworthy that the risk of developing ED appeared to increase with decreasing prostate volume.

**Table 4:** **Significant risk factors for erectile dysfunction after prostate brachytheray in univariate analysis**

	OR ^a^	Standard error	95% CI	p-value
*Age*	1.08	0.023	1.04 – 1.13	**<0.001**
*IIEF score before PB*	0.88	0.026	0.83 – 0.93	**<0.001**
*IIEF classes before PB*				
Severe ED	1.00			
Moderate ED	0.78	0.98	0.06 – 9.19	0.842
Mild to moderate ED	0.17	0.193	0.02 – 1.55	0.117
Mild ED	0.10	0.115	0.01 – 0.91	**0.041**
No DE	0.08	0.085	0.01 – 0.67	**0.021**
*Concern about sexuality*				
No	1.00			
Yes	0.48	0.158	0.25 – 0.91	**0.026**
*Diabetes*				
No	1.00			
Yes	3.47	1.872	1.21 – 9.99	**0.021**
*Prostate volume (cm* ^*3*^ *)*	0.96	0.014	0.94 – 0.99	**0.021**

^a^ OR adjusted by logistic regression for age, PSA level and neoadjuvant androgen deprivation therapy (yes/no).

**Table 5:** **Risk factors for erectile dysfunction after prostate brachytherapy in multivariate analysis**

	OR ^a^	Standard error	95% CI	p-value
*Age*	1.06	0.025	1.02 – 1.12	**<0.001**
*IIEF before PB*	0.89	0.029	0.84 – 0.95	**<0.001**
*Diabetes*				
No	1.00			
Yes	2.60	1.491	0.85 – 8.00	0.094
*Prostate volume (cm3)*	0.96	0.015	0.93 – 0.99	**0.017**
*Concern about sexuality*				
Yes	1.00			
No	0.73	0.263	0.36 – 1.48	0.380

^a^ The logistic regression model contains the binary variable of erection as dependent variable and the variables age, PSA level, hormonal treatment, IIEF score before brachytherapy, diabetes, prostate volume, morning erection and concern about sexuality as independent variables.

### Timing of onset of erectile dysfunction

With regard to the timing of onset of ED, we carried out separate analysis of patients who had received neoadjuvant androgen deprivation therapy. Their time to onset of ED was significantly shorter (3 vs. 16 months.; p = 0.007). Univariate Cox regression confirmed that erectile function deteriorated earlier after neoadjuvant androgen deprivation therapy (HR = 1.97, 95% CI = 1.32 – 2.93, p = 0.0001). In patients who had not had neoadjuvant androgen deprivation therapy, 142 were able to estimate the time to onset of their ED, 30 replied that they had experienced no deterioration and 6 patients were unable to state when erectile function had deteriorated. In patients with diabetes, median time to onset of ED was 4 months compared with 18 months in patients without diabetes (log-rank; p = 0.03). An association was also found between the rapidity of onset of ED and age over 55 years (log-rank; p = 0.01). Cox univariate regression analysis confirmed that the onset of ED after PB was earlier when the patient had diabetes (hazard ratio (HR) = 1.91, 95% CI = 1.12 – 3.25, p = 0.017) or when he was aged over 55 (HR = 0.43, 95% CI = 0.24 – 0.77, p = 0.005).

## Discussion

One of the aims of our study was to clarify the risk of ED after brachytherapy in order to provide patients with fuller information. We observed that even if the onset of erectile deterioration is relatively late (16 months), the majority of patients described a gradual deterioration of erectile function: 73% of patients who had an initial IIEF score greater than 12 downgraded by at least one IIEF score class at a mean follow-up of 36 months. Merrick et al. [14], in a series of 128 patients who were potent before brachytherapy and followed for 29 months, found that 50.5% maintained potency at 3 years. However, several remarks may be made: (1) only one of the participating centers used the 5-point IIEF score for longitudinal follow-up, whereas the other centers used a 4-point score which was considerably lacking in sensitivity, and (2) the authors defined maintenance of potency as an IIEF score of more than 12. This results in a lack of sensitivity, since decreases of the IIEF score above 12 were not taken into account. Another drawback to using reasoning based on a fixed score, rather than in terms of a downgrade in class, is that this makes evaluation dependent on erectile function before treatment. (3) Some of the patients in the study were treated using a modified brachytherapy technique in which the bulb of the penis was spared, with the aim of reducing erectile morbidity. It is therefore difficult to draw from this study conclusion applicable to the general population of patients treated by brachytherapy. In a recent study of Merrick team, in the 226 patients with prostate cancer and preimplant erectile function assessed by IIEF6, the 7-year actuarial rate potency preservation was 55.6% with median postimplant IIEF of 22 in potent patients. Potent patients were statistically younger, had higher preimplant IIEF and were less likely to be diabetic [15]. Potency preservation and median IIEF scores following brachytherapy are durable. Thoughtful dose sparing of proximal penile structures and early penile rehabilitation may further improve these results [15].

It must be noted that most of the patients of our study continued to be sexually active (89%). The present results concern erectile function without any medication, but Schiff et al. previously showed that sildenafil (PDE5 inhibitor) is effective in this population [16]. In our population, only 36 in 171 sexually active men with ED used medication for ED (such as PDE5 inhibitor). These results plead for the development of interventions for a better management of ED in this population [17].

Patients at high risk of ED should be particularly concerned by such interventions. We identified 3 risk factors for ED: preimplant erectile dysfunction, age and prostate volume. Prostate volume was the third significant factor in the multivariate analysis. The patients with a small prostate volume had a high risk of severe ED post PB, independent of the brachytherapy parameters. In our experience, the imaging technique used to calculate prostate volume (MRI for the patients treated in Toulouse) does not seem to modify significantly the performance of the model. Several anatomic structures involved in erectile function may be affected by PB in the case of a small prostate gland, due to an increase risk of radiation outside of the prostate.

Diabetic patients and those treated by neoadjuvant androgen deprivation should also be implicated as they developed early ED. Other factors that could be involved in ED after PB were not explored by the present study: mictional problems, psychological effects related to the fear of cancer, apprehension of contaminating the partner, of pain during intercourse or hematospermia. There is no doubt that such fears could be alleviated by appropriate information.

Moreover, we recently showed that PB resulted in ejaculatory and orgasmic troubles [18]. Taking into account the high prevalence of sexual dysfunction in patients treated by PB, we propose developing supportive interventions in the domain of sexuality to cover the whole pre-treatment and post-treatment period [19].

### Limitations

This is a cohort study and consequently may differ from the overall population of patients treated by PB. However, the criteria of patient inclusion and the treatment modalities used in our centre are widely accepted. Participation rate was high and we verified that participants and men who declined to participate did not differ according to demographic, disease and treatment variables. We do not therefore think that this might be a source of bias.

We cannot rule out the risk of miscalculation related to evaluation by the patient of the date when erectile function began to deteriorate. However, most of the questionnaire uses a validated scale (IIEF-5), which limits the risk of bias. There was only one evaluation of erectile function after PB. This is a shortcoming to study evolution of erectile function in time. Even if our questionnaire is helpful in understanding of the process of occurrence of ED after PB, and taking into account that the mean time of occurrence of ED is late (16 months after PB), questionnaires should be given at standard intervals of 6-12-18-24 months.

In an elderly population (mean age 66 years), an increase of ED incidence with age must be taken into account. The Massachussets Male Aging Study showed that about 10% of the males of the normal population develop ED due to the aging process. However, according to studies in community populations, over a mean period of 3 years the degree of spontaneous deterioration of erectile function should be limited [20].

Factorial analysis showed that an IIEF score of less than 7 after brachytherapy was related to the presence of concurrent disorders: diabetes, obesity, cardiovascular diseases, medical contraindications to surgery and/or neoadjuvant androgen deprivation therapy. However, in multivariate analysis, only age, IIEF score before implantation and prostate volume were found to be significant. Other studies with larger series are certainly required to provide more precise knowledge of risk factors for ED after brachytherapy.

Our evaluation does not take several items into account, such as age of partner or frequency of intercourse. We project to develop a prospective study including these shortcomings for further understanding of this important health related quality of life issue.

## Conclusion

Patients often choose brachytherapy because it is reputed to be less damaging to erectile function. In this study we observed that 36 months after implantation, 73% of men with a IIEF score more than 12 before PB had a deterioration of erectile function by at least one class when measured by the IIEF score. In view of the high prevalence of ED after PB, information before treatment about this morbidity of PB is mandatory, especially in patients at high risk of developing ED (age over 65, preimplant ED and small prostate volume). Our findings contribute further evidence that ED following PB appears progressively after a free interval that may last several months or even years (mean 16 months). Adequate management of ED after treatment should be proposed to patients who wish to maintain sexual function.

## Abbreviations

PB: Prostate brachytherapy
IIEF: International index of erectile function
ED: Erectile dysfunction
Gy: Gray
PSA: Prostate specific antigen
